# Supplementary material for: Distinct prelimbic cortex ensembles encode response execution and inhibition
Source: bioRxiv. 2025 Feb 24:2025.02.23.639736. Preprint. [Version 1] doi: 10.1101/2025.02.23.639736 (PMC11888377; doi:10.1101/2025.02.23.639736)
Supplement: Supplement 1 [file NIHPP2025.02.23.639736v1-supplement-1.pdf]

bioRxiv preprint doi: https://doi.org/10.1101/2025.02.23.639736; this version posted February 24, 2025. The copyright holder for this preprint (which was not certified by peer review) is the author/funder, who has granted bioRxiv a license to display the preprint in perpetuity. It is made available under aCC-BY 4.0 International license.

Supplementary Figures

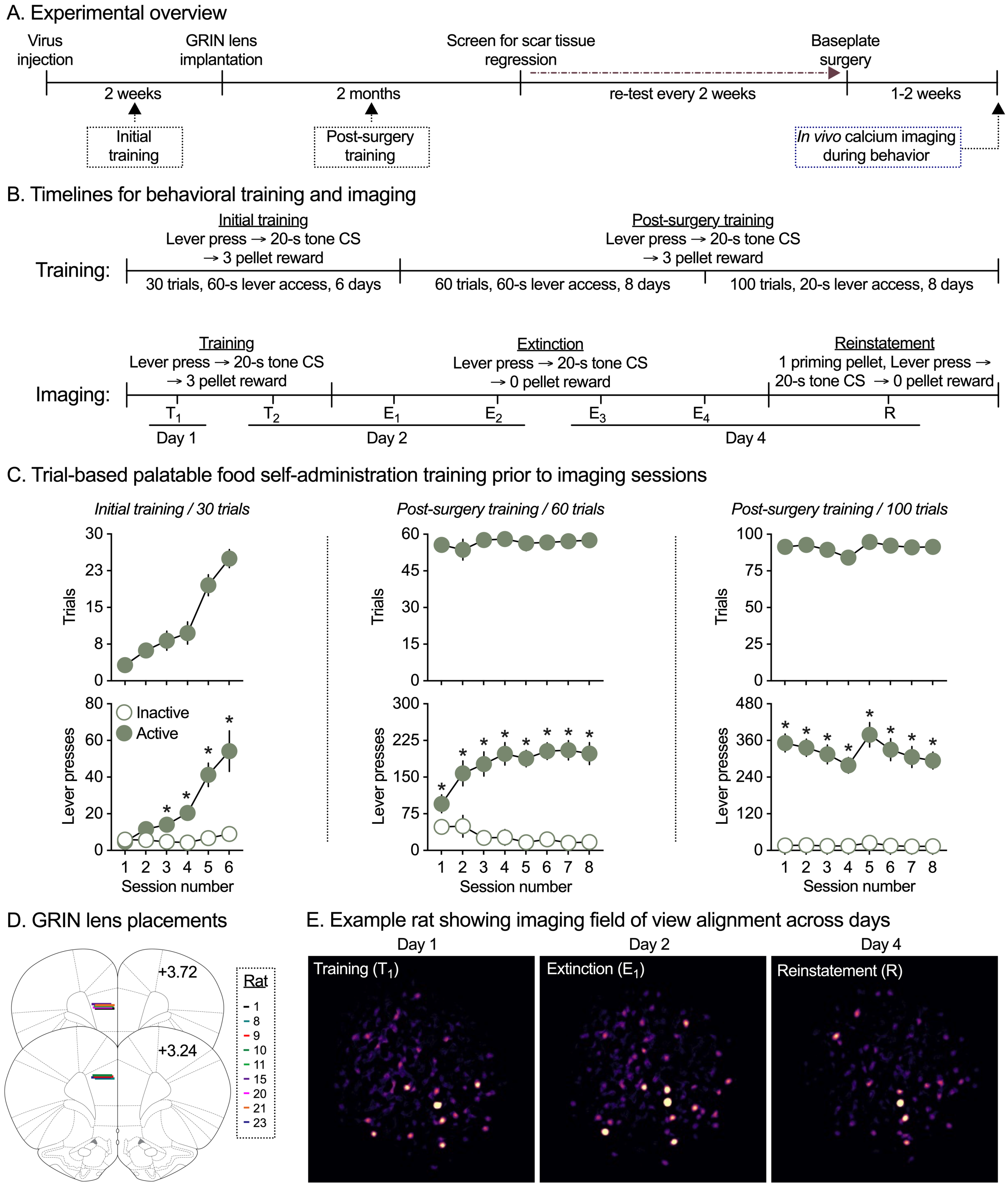

Figure S1. ***In vivo*** **calcium imaging in rats during trial-based palatable food-seeking behavior.**
(A) *Experimental overview.*
(B) *Timeline showing different surgical, behavioral training, and imaging stages.*
(C) *Stages of trial-based palatable food self-administration training.* Rats were trained to self-administer palatable food pellets in three stages consisting of increasing numbers of trials and progressively shorter lever availability periods. Mean (± SEM) number of response trials (top row), and active and inactive lever

presses (bottom row) during each training session for initial training (left panel, 30 trials with 60-s lever access/trial), post-surgery training phase 1 (center panel, 60 trials with 60-s lever access/trial) and post-surgery training phase 2 (right panel, 100 trials with 20-s lever access/trial). *Significant difference ($p < 0.05$) between active and inactive lever presses during a training session.
(D) *GRIN lens placements.* Lines represent location of the base of imaging GRIN lens for rats included in the study (n = 9).
(E) *Representative imaging field of view aligned across imaging days.* Maximum intensity projection of trial-wise correlation x standard deviation from one example rat to show alignment across sessions and over four imaging days.

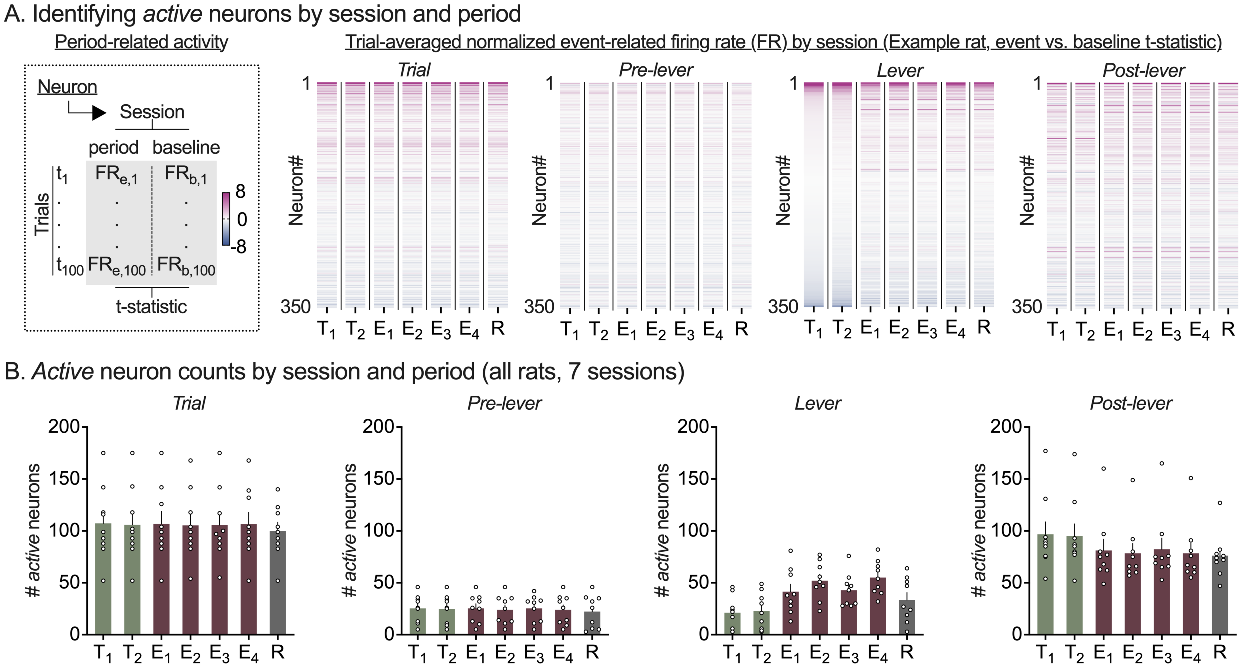

Figure S2. PL active neuron identification and overlap across all 7 imaging sessions.
(A) *Active neuron identification within a trial period.* For each rat, spikes were estimated from calcium transients and used to calculate trial-by-trial average firing rate of individual neurons (all neurons detected across all 7 sessions) within each trial period. Next, for each session and trial period, *active* neurons were identified as those with significantly different average firing rate during that period vs. period at the start of the same trial in the session. Heatmaps showing t-statistic (period vs. baseline) of all neurons from one example rat for the entire trial (left), pre-lever period (center left), lever availability period (center right) and post-lever period (right). For all heatmaps, columns represent imaging session, and rows represent individual neurons ranked by t-statistic value for lever period activity in training session 1.
(B) *Active neuron counts by trial period and imaging session.* Mean (± SEM) number of *active* neurons identified during the entire trial (left), pre-lever period (center left), lever availability period (center right) and post-lever period (right), for each imaging session. Clear circles represent data from individual rats (n = 9).

bioRxiv preprint doi: https://doi.org/10.1101/2025.02.23.639736; this version posted February 24, 2025. The copyright holder for this preprint (which was not certified by peer review) is the author/funder, who has granted bioRxiv a license to display the preprint in perpetuity. It is made available under aCC-BY 4.0 International license.

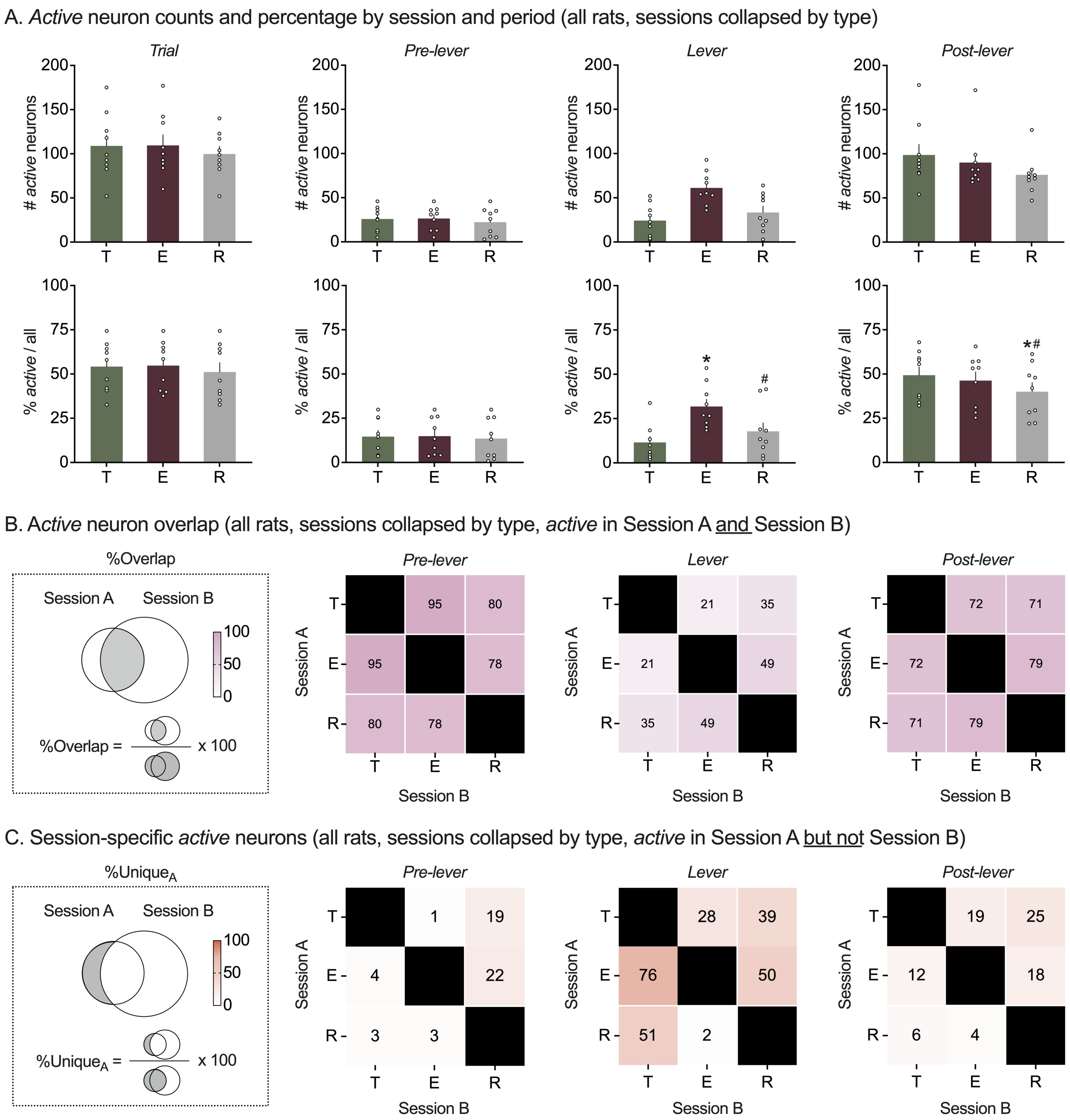

Figure S3. PL active neuron counts and overlap across 3 imaging session types (within trial period type).
(A) *Active neuron identification trial period and imaging session. For each rat,* active neurons were detected within each trial period and for all 7 imaging sessions as described earlier and populations were then collapsed by 3 imaging session types (training, extinction or reinstatement). Mean (± SEM) number (top) and percentage (bottom) of *active* neurons identified during the entire trial (left), pre-lever period (center left), lever availability period (center right) and post-lever period (right), for each imaging session. Clear circles represent data from individual rats (n = 9).
(B) Shared *active PL neurons across 3 imaging session types.* For each rat, within a trial period and session pair, the percentage of overlapping *active* neurons was calculated as shown in the schematic (left). Heatmaps show mean pairwise %overlapping neurons for pre-lever (left), lever period (middle) and post-lever period (right), for the 3 imaging session types.

bioRxiv preprint doi: https://doi.org/10.1101/2025.02.23.639736; this version posted February 24, 2025. The copyright holder for this preprint (which was not certified by peer review) is the author/funder, who has granted bioRxiv a license to display the preprint in perpetuity. It is made available under aCC-BY 4.0 International license.

(C) *Session-specific active PL neurons across 3 imaging session types.* For each rat, within a trial period and session type pair, the percentage of session-specific *active* neurons was calculated as shown in the schematic (left). Heatmaps show mean pairwise %unique neurons for pre-lever (left), lever period (middle) and post-lever period (right), for the 3 imaging session types.

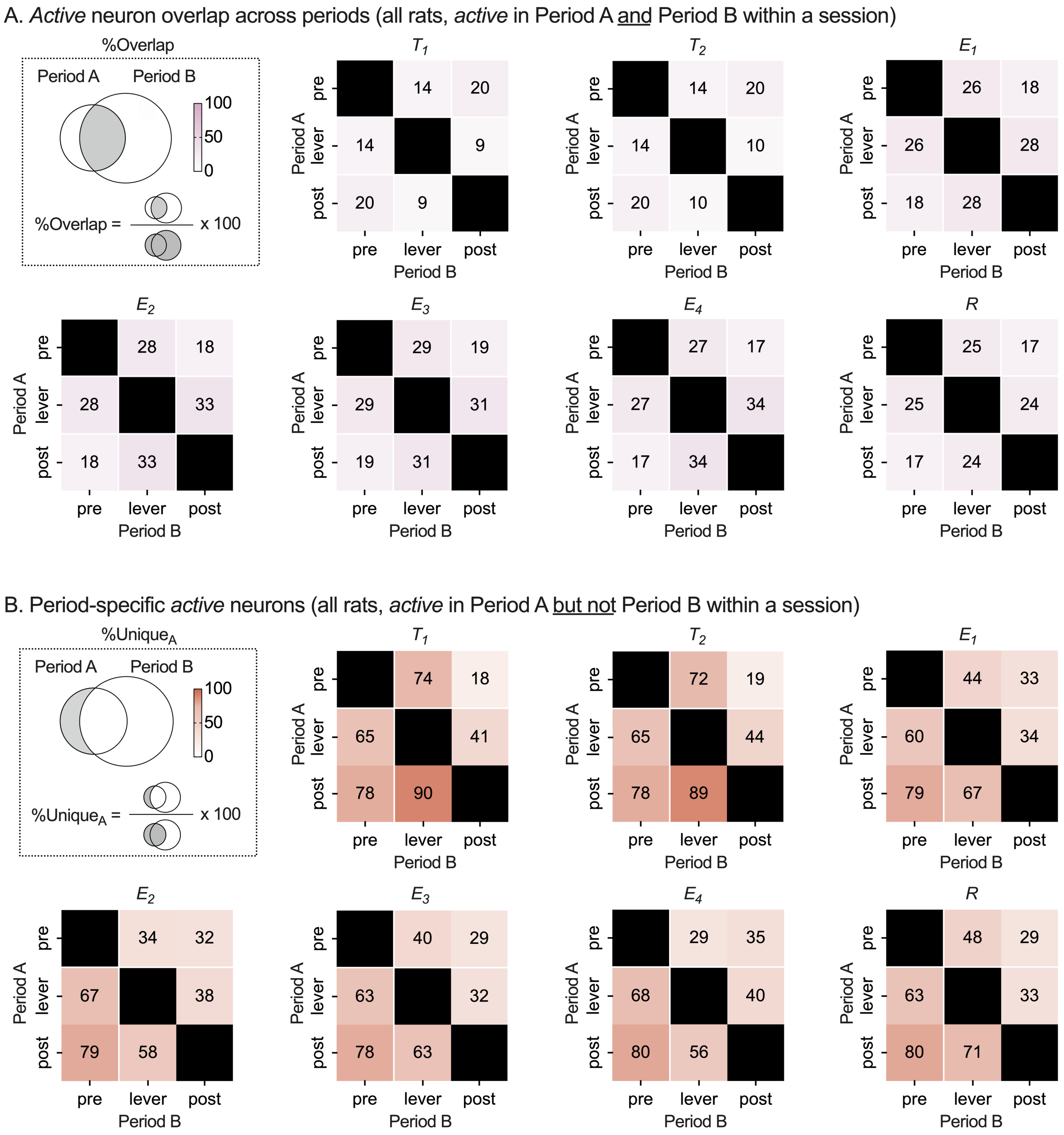

Figure S4. Shared and specific PL active neurons across trial periods (within imaging session).
(A) *Active neuron overlap across trial periods. For each rat,* active neurons were detected within each trial period and for all 7 imaging sessions as described earlier. Next, within a session, percentage *active* neuron overlap was calculated pairwise between the three trial periods (pre-level, lever, post-lever) as shown in the schematic (left). Heatmaps show mean pairwise %overlap for each imaging session (7 sessions).
(B) Period-*specific active PL neurons.* For each rat, within a trial period pair, the percentage of period-specific *active* neurons was calculated as shown in the schematic (left). Heatmaps show mean pairwise %unique neurons for each imaging session (7 sessions).

bioRxiv preprint doi: https://doi.org/10.1101/2025.02.23.639736; this version posted February 24, 2025. The copyright holder for this preprint (which was not certified by peer review) is the author/funder, who has granted bioRxiv a license to display the preprint in perpetuity. It is made available under aCC-BY 4.0 International license.

A. Response/no-response classification accuracy during training and extinction sessions

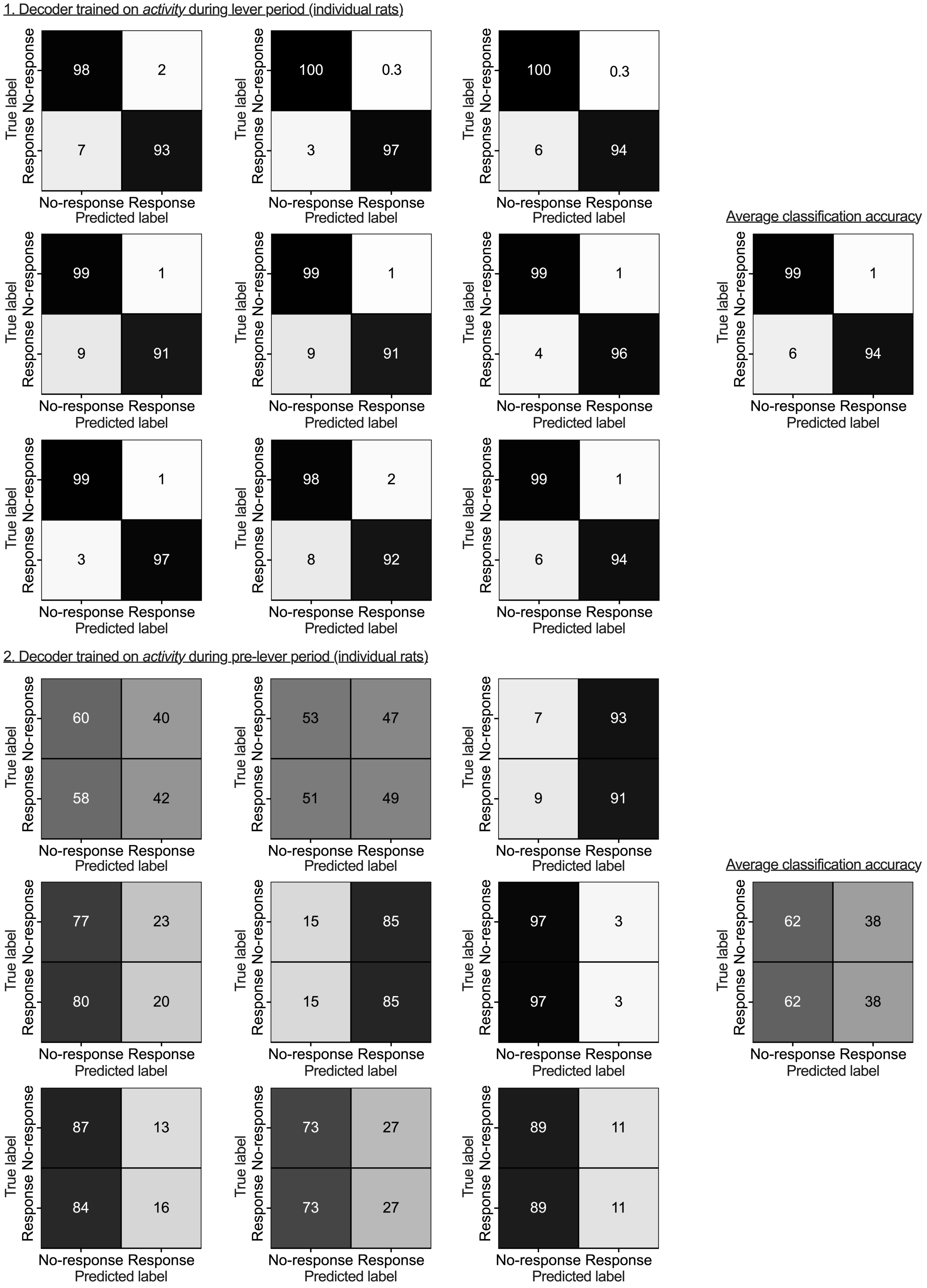

bioRxiv preprint doi: https://doi.org/10.1101/2025.02.23.639736; this version posted February 24, 2025. The copyright holder for this preprint (which was not certified by peer review) is the author/funder, who has granted bioRxiv a license to display the preprint in perpetuity. It is made available under aCC-BY 4.0 International license.

Figure S5. Individual rat lever period and pre-lever period decoder accuracy during training and extinction sessions

(A.1) Lever period decoder: For each rat, a binary (response/no-response) linear decoder was trained using the average firing rate vector during lever availability period, for a randomly selected subset of training and extinction trials (75/25 stratified split between training and test trials) and tested on the holdout subset of training and extinction trials. Response/no-response classification accuracy on the holdout test set (training and extinction) split by response and shown as a confusion matrix for individual rats (left 3 x 3 heatmap grid) or averaged across all rats (right heatmap, *n* = 9).

(A.2) Pre-lever period decoder: For each rat, a binary (response/no-response) linear decoder was trained using the average firing rate vector during pre-lever availability period, for a randomly selected subset of training and extinction trials (75/25 stratified split between training and test trials) and tested on the holdout subset of training and extinction trials. Response/no-response classification accuracy on the holdout test set (training and extinction) split by response and shown as a confusion matrix for individual rats (left 3 x 3 heatmap grid) or averaged across all rats (right heatmap, *n* = 9).

bioRxiv preprint doi: https://doi.org/10.1101/2025.02.23.639736; this version posted February 24, 2025. The copyright holder for this preprint (which was not certified by peer review) is the author/funder, who has granted bioRxiv a license to display the preprint in perpetuity. It is made available under aCC-BY 4.0 International license.

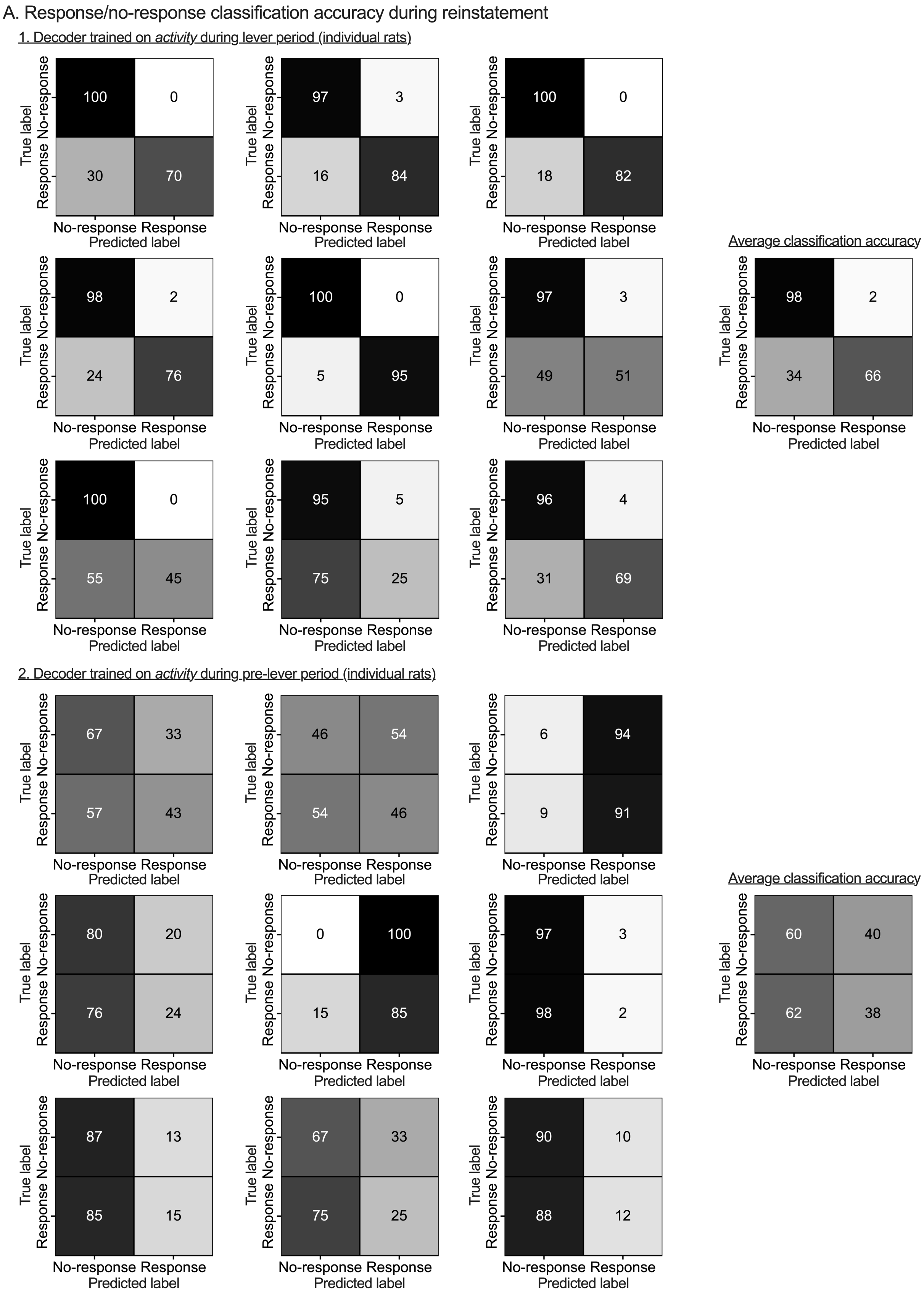

bioRxiv preprint doi: https://doi.org/10.1101/2025.02.23.639736; this version posted February 24, 2025. The copyright holder for this preprint (which was not certified by peer review) is the author/funder, who has granted bioRxiv a license to display the preprint in perpetuity. It is made available under aCC-BY 4.0 International license.

Figure S6. Individual rat lever period and pre-lever period decoder accuracy during the reinstatement session

(A.1) Lever period decoder: For each rat, a binary (response/no-response) linear decoder was trained using the average firing rate vector during lever availability period, for a randomly selected subset of training and extinction trials (75/25 stratified split between training and test trials) and tested on all reinstatement session trials. Response/no-response classification accuracy during the reinstatement session split by response and shown as a confusion matrix for individual rats (left 3 x 3 heatmap grid) or averaged across all rats (right heatmap, *n* = 9).

(A.2) Pre-lever period decoder: For each rat, a binary (response/no-response) linear decoder was trained using the average firing rate vector during pre-lever availability period, for a randomly selected subset of training and extinction trials (75/25 stratified split between training and test trials) and tested on all reinstatement session trials. Response/no-response classification accuracy on the reinstatement session split by response and shown as a confusion matrix for individual rats (left 3 x 3 heatmap grid) or averaged across all rats (right heatmap, *n* = 9).

bioRxiv preprint doi: https://doi.org/10.1101/2025.02.23.639736; this version posted February 24, 2025. The copyright holder for this preprint (which was not certified by peer review) is the author/funder, who has granted bioRxiv a license to display the preprint in perpetuity. It is made available under aCC-BY 4.0 International license.

Supplementary tables

Table S1. Detailed statistical outputs for behavior during training and imaging sessions.

Experimental phase	Behavioral measure	Factors in analysis	Statistical output			Figure
Initial training / 30 trials per session	Trials (*n* = 9)	RM-ANOVA (Session)	F-value	*p*-value	Partial η2	S1C, left panel
		Session (within-subjects)	F(3.359,26.873) = 28.070	< 0.001	0.778	
	Lever presses (*n* = 9)	RM-ANOVA (Lever x Session)	F-value	*p*-value	Partial η2	S1C, left panel
		Lever (within-subjects)	F(1,8) = 37.110	< 0.001	0.823	
		Session (within-subjects)	F(1.653,13.221) = 10.991	0.002	0.579	
		Lever x Session	F(1.828,14.624) = 12.849	< 0.001	0.410	
		Bonferroni (Active lever vs. Inactive lever)	Mean Difference (Group 1 - Group 2)	Std. Error	*p*-value	
		Session 1	-1.222	2.385	0.622	
		Session 2	6	3.460	0.121	
		Session 3	9.444	2.729	0.009	
		Session 4	16.111	3.525	0.002	
		Session 5	34.444	7.153	0.001	
		Session 6	45.222	9.500	0.001	
Post-surgery training phase 1 / 60 trials per session	Trials (*n* = 9)	RM-ANOVA (Session)	F-value	*p*-value	Partial η2	S1C, center panel
		Session (within-subjects)	F(1.705,13.644) = 0.735	0.643	0.084	
	Lever presses (*n* = 9)	RM-ANOVA (Lever x Session)	F-value	*p*-value	Partial η2	S1C, center panel
		Lever (within-subjects)	F(1, 8) = 58.556	< 0.001	0.880	
		Session (within-subjects)	F(2.513, 20.106) = 2.258	0.121	0.220	
		Lever x Session	F(3.201, 25.611) = 9.797	< 0.001	0.550	

bioRxiv preprint doi: https://doi.org/10.1101/2025.02.23.639736; this version posted February 24, 2025. The copyright holder for this preprint (which was not certified by peer review) is the author/funder, who has granted bioRxiv a license to display the preprint in perpetuity. It is made available under aCC-BY 4.0 International license.

		Bonferroni (Active lever vs. Inactive lever)	Mean Difference (Group 1 - Group 2)	Std. Error	*p*-value	
		Session 1	47.333	18.880	0.037	
		Session 2	108.000	31.153	0.008	
		Session 3	151.222	28.017	< 0.001	
		Session 4	170.889	27.646	< 0.001	
		Session 5	171.222	16.740	< 0.001	
		Session 6	180.556	20.759	< 0.001	
		Session 7	189.222	22.589	< 0.001	
		Session 8	181.444	25.994	< 0.001	
Post-surgery training phase 2 / 100 trials per session	Trials (*n* = 9)	RM-ANOVA (Session)	F-value	*p*-value	Partial η2	S1C, right panel
		Session (within-subjects)	F(2.942,23.540) = 2.869	0.012	0.264	
	Lever presses (*n* = 9)	RM-ANOVA (Lever x Session)	F-value	*p*-value	Partial η2	S1C, right panel
		Lever (within-subjects)	F(1,8) = 110.338	< 0.001	0.932	
		Session (within-subjects)	F(2.959,23.674) = 4.202	0.016	0.344	
		Lever x Session	F(4.095,32.764) = 4.931	0.003	0.381	
		Bonferroni (Active lever vs. Inactive lever)	Mean Difference (Group 1 - Group 2)	Std. Error	*p*-value	
		Session 1	335.889	29.592	< 0.001	
		Session 2	319.111	26.907	< 0.001	
		Session 3	299.556	30.210	< 0.001	
		Session 4	263.556	26.558	< 0.001	
		Session 5	353.889	41.535	< 0.001	
		Session 6	314.444	34.590	< 0.001	
		Session 7	292.444	34.060	< 0.001	
		Session 8	280.000	28.123	< 0.001	
Imaging sessions / 100 trials per session	Trials (*n* = 9)	RM-ANOVA (Session)	F-value	*p*-value	Partial η2	1B
		Session (within-subjects)	F(2.410,19.284) = 83.046	< 0.001	0.912	
		Bonferroni (Active lever, vs. Training 1)	Mean Difference (Group 1 - Group 2)	Std. Error	*p*-value	
		Training 1	-	-	-	
		Training 2	0.333	0.707	1.000	

bioRxiv preprint doi: https://doi.org/10.1101/2025.02.23.639736; this version posted February 24, 2025. The copyright holder for this preprint (which was not certified by peer review) is the author/funder, who has granted bioRxiv a license to display the preprint in perpetuity. It is made available under aCC-BY 4.0 International license.

		Extinction 1	57.222	6.087	< 0.001	
		Extinction 2	81.556	4.062	< 0.001	
		Extinction 3	61.000	4.059	< 0.001	
		Extinction 4	88.000	1.756	< 0.001	
		Reinstatement	36.222	6.843	0.015	
		Bonferroni (Active lever, vs. Extinction 4)	Mean Difference (Group 1 - Group 2)	Std. Error	*p*-value	
		Training 1	-88.000	1.755942	< 0.001	
		Training 2	-87.667	1.724	< 0.001	
		Extinction 1	-30.778	6.166	0.022	
		Extinction 2	-6.444	3.783	1.000	
		Extinction 3	-27.000	4.301	0.005	
		Extinction 4	-	-	-	
		Reinstatement	-51.778	6.159	< 0.001	
	Lever presses (*n* = 9)	RM-ANOVA (Lever x Session)	F-value	*p*-value	Partial η2	1B
		Lever (within-subjects)	F(1,7) = 114.653	< 0.001	0.942	
		Session (within-subjects)	F(1.365,9.557) = 19.402	< 0.001	0.735	
		Lever x Session	F(1.288,9.017) = 18.749	0.001	0.728	
		Bonferroni (Active lever vs. Inactive lever)	Mean Difference (Group 1 - Group 2)	Std. Error	*p*-value	
		Training 1	263.875	35.286	< 0.001	
		Training 2	285.625	50.750	< 0.001	
		Extinction 1	96.375	15.361	< 0.001	
		Extinction 2	30.375	8.797	0.011	
		Extinction 3	78.500	12.252	< 0.001	
		Extinction 4	17.625	4.694	0.007	
		Reinstatement	132.500	19.147	< 0.001	
		Bonferroni (Active lever, vs. Training 1)	Mean Difference (Group 1 - Group 2)	Std. Error	*p*-value	
		Training 1	-	-	-	
		Training 2	-22.125	24.693	1.000	
		Extinction 1	164.875	47.672	0.222	
		Extinction 2	229.500	36.017	0.008	
		Extinction 3	182.500	37.469	0.038	

bioRxiv preprint doi: https://doi.org/10.1101/2025.02.23.639736; this version posted February 24, 2025. The copyright holder for this preprint (which was not certified by peer review) is the author/funder, who has granted bioRxiv a license to display the preprint in perpetuity. It is made available under aCC-BY 4.0 International license.

		Extinction 4	244.000	35.643	0.005	
		Reinstatement	126.500	22.415	0.016	
		Bonferroni (Active lever, vs. Extinction 4)	Mean Difference (Group 1 - Group 2)	Std. Error	*p*-value	
		Training 1	-244.000	35.643	0.005	
		Training 2	-266.125	51.922	0.029	
		Extinction 1	-79.125	13.938	0.016	
		Extinction 2	-14.500	5.922	0.928	
		Extinction 3	-61.500	9.914	0.009	
		Extinction 4	-	-	-	
		Reinstatement	-117.500	18.234	0.007	

Table S2. Detailed statistical outputs for analyses pertaining to number and percentage of active neurons by session (7 sessions) and trial period

**Measure**	**Factors in analysis**	**Statistical output**			**Figure**
Percent active (*n* = 9)	RM-ANOVA (7 Sessions x 3 Periods)	F-value	P-value	Partial η2	1D
	Period (within-subjects)	F(1.414,11.310) = 38.628	< 0.001	0.828	
	Session (within-subjects)	F(2.240,17.922) = 4.228	0.028	0.346	
	Period x Session	F(2.734,21.873) = 15.629	< 0.001	0.661	
Percent active during trial (*n* = 9)	RM-ANOVA (Session)	F-value	*p*-value	Partial η2	1D, panel 1
	Session (within-subjects)	F(1.938,15.506) = 1.888	0.185	0.191	
Percent active during pre-lever period (*n* = 9)	RM-ANOVA (Session)	F-value	*p*-value	Partial η2	1D, panel 2
	Session (within-subjects)	F(1.874,14.990) = 1.013	0.382	0.112	
Percent active during lever period (*n* = 9)	RM-ANOVA (Session)	F-value	*p*-value	Partial η2	1D, panel 3
	Session (within-subjects)	F(2.320,18.561) = 13.157	< 0.001	0.622	
	Bonferroni (%active in lever period, vs. Training 1)	Mean Difference (Group 1 - Group 2)	Std. Error	*p*-value	
	Training 1	-	-	-	
	Training 2	-0.698	0.544	1.000	

bioRxiv preprint doi: https://doi.org/10.1101/2025.02.23.639736; this version posted February 24, 2025. The copyright holder for this preprint (which was not certified by peer review) is the author/funder, who has granted bioRxiv a license to display the preprint in perpetuity. It is made available under aCC-BY 4.0 International license.

	Extinction 1	-10.624	2.952	0.147	
	Extinction 2	-16.562	2.850	0.008	
	Extinction 3	-12.588	3.025	0.066	
	Extinction 4	-18.876	3.585	0.016	
	Reinstatement	-7.665	2.875	0.599	
	Bonferroni (%active in lever period, vs. Extinction 4)	Mean Difference (Group 1 - Group 2)	Std. Error	*p*-value	
	Training 1	18.876	3.585	0.016	
	Training 2	18.178	3.843	0.031	
	Extinction 1	8.252	2.906	0.458	
	Extinction 2	2.314	1.550	1.000	
	Extinction 3	6.288	1.077	0.008	
	Extinction 4	-	-	-	
	Reinstatement	11.212	2.834	0.088	
Percent active during post-lever period (*n* = 9)	RM-ANOVA (Session)	F-value	*p*-value	Partial η2	1D, panel 4
	Session (within-subjects)	F(2.838,22.701) = 11.376	< 0.001	0.587	
	Bonferroni (%active in post-lever period, vs. Training 1)	Mean Difference (Group 1 - Group 2)	Std. Error	*p*-value	
	Training 1	-	-	-	
	Training 2	0.480	0.732	1.000	
	Extinction 1	7.127	1.053	0.003	
	Extinction 2	8.471	1.689	0.022	
	Extinction 3	6.255	2.017	0.308	
	Extinction 4	8.553	1.634	0.017	
	Reinstatement	8.327	1.973	0.061	
	Bonferroni (%active in post-lever period, vs. Extinction 4)	Mean Difference (Group 1 - Group 2)	Std. Error	*p*-value	
	Training 1	-8.553	1.634	0.017	
	Training 2	-8.073	1.658	0.026	
	Extinction 1	-1.426	1.072	1.000	
	Extinction 2	-0.082	0.694	1.000	
	Extinction 3	-2.298	1.192	1.000	
	Extinction 4	-	-	-	
	Reinstatement	-0.226	2.038	1.000	

bioRxiv preprint doi: https://doi.org/10.1101/2025.02.23.639736; this version posted February 24, 2025. The copyright holder for this preprint (which was not certified by peer review) is the author/funder, who has granted bioRxiv a license to display the preprint in perpetuity. It is made available under aCC-BY 4.0 International license.

Table S3. Detailed statistical outputs for analyses pertaining to number and percentage of active neurons by session-type (3 session types) and trial period

Measure	Factors in analysis	Statistical output			Figure
Percent active during trial (*n* = 9)	RM-ANOVA (3 Session types x 3 Periods)	F-value	*p*-value	Partial η2	S3A
	Period (within-subjects)	F(1.494,11.956) = 37.606	< 0.001	0.825	
	Session type (within-subjects)	F(1.304,10.429) = 18.018	< 0.001	0.693	
	Period x Session type	F(2.325,18.599) = 18.933	< 0.001	0.703	
Percent active during trial (*n* = 9)	RM-ANOVA (Session type)	F-value	*p*-value	Partial η2	S3A, panel 1
	Session (within-subjects)	F(1.889,15.111) = 3.888	0.042	0.327	
	Bonferroni (%active in trial, vs. Training)	Mean Difference (Group 1 - Group 2)	Std. Error	*p*-value	
	Training	-	-	-	
	Extinction	-0.580	1.243	1.000	
	Reinstatement	3.000	1.527	0.255	
	Bonferroni (%active in trial, vs. Extinction)	Mean Difference (Group 1 - Group 2)	Std. Error	*p*-value	
	Training	0.580	1.243	1.000	
	Extinction	-	-	-	
	Reinstatement	3.580	1.350	0.087	
Percent active during pre-lever period (*n* = 9)	RM-ANOVA (Session type)	F-value	*p*-value	Partial η2	
	Session (within-subjects)	F(1.437,11.499) = 3.139	0.071	0.282	S3A, panel 2
Percent active during lever period (*n* = 9)	RM-ANOVA (Session type)	F-value	*p*-value	Partial η2	S3A, panel 3
	Session (within-subjects)	F(1.679,13.433) = 22.626	< 0.001	0.739	
	Bonferroni (%active in lever period, vs. Training)	Mean Difference (Group 1 - Group 2)	Std. Error	*p*-value	
	Training	-	-	-	
	Extinction	-20.194	3.635	0.002	
	Reinstatement	-6.173	3.018	0.225	
	Bonferroni (%active in lever period, vs. Extinction)	Mean Difference (Group 1 - Group 2)	Std. Error	*p*-value	
	Training	-20.194	3.635	0.002	

bioRxiv preprint doi: https://doi.org/10.1101/2025.02.23.639736; this version posted February 24, 2025. The copyright holder for this preprint (which was not certified by peer review) is the author/funder, who has granted bioRxiv a license to display the preprint in perpetuity. It is made available under aCC-BY 4.0 International license.

	Extinction	-	-	-	
	Reinstatement	14.020	2.465	0.001	
Percent active during post-lever period (*n* = 9)	RM-ANOVA (Session type)	F-value	*p*-value	Partial η2	S3A, panel 4
	Session (within-subjects)	F(1.947,15.574) = 10.909	0.001	0.577	
	Bonferroni (%active in pos-lever period, vs. Training)	Mean Difference (Group 1 - Group 2)	Std. Error	*p*-value	
	Training	-	-	-	
	Extinction	3.077	2.170	0.582	
	Reinstatement	9.314	1.877	0.003	
	Bonferroni (%active in post-lever period, vs. Extinction)	Mean Difference (Group 1 - Group 2)	Std. Error	*p*-value	
	Training	3.077	2.170	0.582	
	Extinction	-	-	-	
	Reinstatement	6.238	2.038	0.047	

Table S4. Detailed statistical outputs for trial-wise decoder accuracy during training and extinction sessions

**Measure**	**Factors in analysis**	**Statistical output**			**Figure**
Decoder accuracy on Training and Extinction trials (*n* = 9)	RM-ANOVA (Drop type x Population)	F-value	*p*-value	Partial η2	2B.2, left
	Population (within-subjects)	F(1.502,12.015) = 12.798	0.002	0.615	
	Drop type (within-subjects)	F(1,8) = 26.350	0.001	0.767	
	Drop type x Population	F(1.964,15.709) = 2.547	0.043	0.241	
	Bonferroni (*Active* vs. Random)	Mean Difference (Group 1 - Group 2)	Std. Error	*p*-value	
	All training (T)	-0.102	0.048	0.067	
	All extinction (E)	-0.129	0.041	0.013	
	All (T∪E)	-0.174	0.029	0.000	
	Overlap (T∩E)	-0.102	0.040	0.034	
	Only Training (T-E)	-0.021	0.013	0.140	
	Only Extinction (E-T)	-0.109	0.030	0.006	

bioRxiv preprint doi: https://doi.org/10.1101/2025.02.23.639736; this version posted February 24, 2025. The copyright holder for this preprint (which was not certified by peer review) is the author/funder, who has granted bioRxiv a license to display the preprint in perpetuity. It is made available under aCC-BY 4.0 International license.

Table S5. Detailed statistical outputs for trial-wise decoder accuracy during training and extinction following exclusion of specific active neuron sub-populations

Measure	*Active* neuron population	Factors in analysis	Statistical output			Figure
Decoder accuracy on Training and Extinction trials (n = 9)	All training (T)	RM-ANOVA (Response x Drop type)	F-value	*p*-value	Partial η2	2B.3, top left
		Response (within-subjects)	F(1,8) = 6.209	0.037	0.437	
		Drop type (within-subjects)	F(1,8) = 3.977	0.081	0.332	
		Response x Drop type	F(1,8) = 6.088	0.039	0.432	
		Bonferroni (*Active* vs. Random)	Mean Difference (Group 1 - Group 2)	Std. Error	*p*-value	
		Response	-0.243	0.108	0.055	
		No-response	0.028	0.010	0.020	
	All extinction (E)	RM-ANOVA (Response x Drop type)	F-value	*p*-value	Partial η2	2B.3, bottom left
		Response (within-subjects)	F(1,8) = 0.434	0.529	0.051	
		Drop type (within-subjects)	F(1,8) = 10.403	0.012	0.565	
		Response x Drop type	F(1,8) = 1.126	0.320	0.123	
		Bonferroni (*Active* vs. Random)	Mean Difference (Group 1 - Group 2)	Std. Error	*p*-value	
		Response	-0.059	0.092	0.540	
		No-response	-0.246	0.108	0.051	
	Only Training (T-E)	RM-ANOVA (Response x Drop type)	F-value	*p*-value	Partial η2	2B.3, top center
		Response (within-subjects)	F(1,8) = 0.278	0.612	0.034	
		Drop type (within-subjects)	F(1,8) = 2.993	0.122	0.272	
		Response x Drop type	F(1,8) = 1.901	0.205	0.192	
		Bonferroni (*Active* vs. Random)	Mean Difference (Group 1 - Group 2)	Std. Error	*p*-value	
		Response	0.011	0.015	0.508	
		No-response	-0.053	0.033	0.153	
	Only Extinction (E-T)	RM-ANOVA (Response x Drop type)	F-value	*p*-value	Partial η2	2B.3, bottom center
		Response (within-subjects)	F(1,8) = 9.909	0.014	0.553	
		Drop type (within-subjects)	F(1,8) = 11.554	0.009	0.591	
		Response x Drop type	F(1,8) = 45.604	< 0.001	0.851	
		Bonferroni (*Active* vs. Random)	Mean Difference (Group 1 - Group 2)	Std. Error	*p*-value	

bioRxiv preprint doi: https://doi.org/10.1101/2025.02.23.639736; this version posted February 24, 2025. The copyright holder for this preprint (which was not certified by peer review) is the author/funder, who has granted bioRxiv a license to display the preprint in perpetuity. It is made available under aCC-BY 4.0 International license.

		Response	0.124	0.006	<0.001	
		No-response	-0.374	0.073	<0.001	
	Either T or E (T∪E)	RM-ANOVA (Response x Drop type)	F-value	*p*-value	Partial η2	2B.3, top right
		Response (within-subjects)	F(1,8) = 0.450	0.521	0.053	
		Drop type (within-subjects)	F(1,8) = 35.571	<0.001	0.816	
		Response x Drop type	F(1,8) = 1.455	0.262	0.154	
		Bonferroni (*Active* vs. Random)	Mean Difference (Group 1 - Group 2)	Std. Error	*p*-value	
		Response	-0.075	0.105	0.497	
		No-response	-0.326	0.114	0.021	
	Both T & E (T∩E)	RM-ANOVA (Response x Drop type)	F-value	*p*-value	Partial η2	2B.3, bottom right
		Response (within-subjects)	F(1,8) = 12.586	0.008	0.611	
		Drop type (within-subjects)	F(1,8) = 4.787	0.060	0.374	
		Response x Drop type	F(1,8) = 9.020	0.017	0.530	
		Bonferroni (*Active* vs. Random)	Mean Difference (Group 1 - Group 2)	Std. Error	*p*-value	
		Response	-0.254	0.095	0.029	
		No-response	0.044	0.021	0.075	

Table S6. Detailed statistical outputs for trial-wise decoder accuracy during reinstatement

Measure	Factors in analysis	Statistical output			Figure
Decoder accuracy on Reinstatement trials (*n* = 9)	RM-ANOVA (Drop type x Population)	F-value	*p*-value	Partial η2	3B.2, left
	Population (within-subjects)	F(1.636,13.091) = 3.848	0.006	0.325	
	Drop type (within-subjects)	F(1,8) = 2.704	0.139	0.253	
	Drop type x Population	F(2.040,16.317) = 2.872	0.085	0.264	
	Bonferroni (*Active* vs. Random)	Mean Difference (Group 1 - Group 2)	Std. Error	*p*-value	
	All training (T)	-0.071	0.033	0.061	
	All extinction (E)	-0.064	0.030	0.069	
	All (T∪E)	-0.035	0.050	0.506	
	Overlap (T∩E)	-0.097	0.029	0.011	
	Only Training (T-E)	0.030	0.024	0.248	
	Only Extinction (E-T)	0.006	0.038	0.888	

bioRxiv preprint doi: https://doi.org/10.1101/2025.02.23.639736; this version posted February 24, 2025. The copyright holder for this preprint (which was not certified by peer review) is the author/funder, who has granted bioRxiv a license to display the preprint in perpetuity. It is made available under aCC-BY 4.0 International license.

Table S7. Detailed statistical outputs for trial-wise decoder accuracy during reinstatement following exclusion of specific active neuron sub-populations

Measure	*Active* neuron population	Factors in analysis	Statistical output			Figure
Decoder accuracy on Reinstatement trials (n = 9)	All training (T)	RM-ANOVA (Response x Drop type)	F-value	*p*-value	Partial η2	3B.3, top left
		Response (within-subjects)	F(1,8) = 16.358	0.004	0.672	
		Drop type (within-subjects)	F(1,8) = 3.726	0.090	0.032	
		Response x Drop type	F(1,8) = 5.228	0.052	0.395	
		Bonferroni (*Active* vs. Random)	Mean Difference (Group 1 - Group 2)	Std. Error	*p*-value	
		Response	-0.152	0.069	0.058	
		No-response	0.024	0.020	0.267	
	All extinction (E)	RM-ANOVA (Response x Drop type)	F-value	*p*-value	Partial η2	3B.3, bottom left
		Response (within-subjects)	F(1,8) = 0.227	0.646	0.028	
		Drop type (within-subjects)	F(1,8) = 9.482	0.015	0.542	
		Response x Drop type	F(1,8) = 2.830	0.131	0.261	
		Bonferroni (*Active* vs. Random)	Mean Difference (Group 1 - Group 2)	Std. Error	*p*-value	
		Response	0.010	0.067	0.881	
		No-response	-0.272	0.115	0.045	
	Only Training (T-E)	RM-ANOVA (Response x Drop type)	F-value	*p*-value	Partial η2	3B.3, top center
		Response (within-subjects)	F(1,8) = 23.942	0.001	0.750	
		Drop type (within-subjects)	F(1,8) = 0.523	0.490	0.061	
		Response x Drop type	F(1,8) = 2.625	0.144	0.247	
		Bonferroni (*Active* vs. Random)	Mean Difference (Group 1 - Group 2)	Std. Error	*p*-value	
		Response	0.058	0.037	0.161	
		No-response	-0.042	0.027	0.157	
	Only Extinction (E-T)	RM-ANOVA (Response x Drop type)	F-value	*p*-value	Partial η2	3B.3, bottom center
		Response (within-subjects)	F(1,8) = 0.346	0.573	0.041	
		Drop type (within-subjects)	F(1,8) = 2.871	0.129	0.264	

bioRxiv preprint doi: https://doi.org/10.1101/2025.02.23.639736; this version posted February 24, 2025. The copyright holder for this preprint (which was not certified by peer review) is the author/funder, who has granted bioRxiv a license to display the preprint in perpetuity. It is made available under aCC-BY 4.0 International license.

		Response x Drop type	F(1,8) = 93.351	< 0.001	0.921	
		Bonferroni (*Active* vs. Random)	Mean Difference (Group 1 - Group 2)	Std. Error	*p*-value	
		Response	0.229	0.036	< 0.001	
		No-response	-0.408	0.080	< 0.001	
	All (T∪E)	RM-ANOVA (Response x Drop type)	F-value	*p*-value	Partial η2	3B.3, top right
		Response (within-subjects)	F(1,8) = 0.015	0.904	0.002	
		Drop type (within-subjects)	F(1,8) = 13.156	0.007	0.622	
		Response x Drop type	F(1,8) = 3.704	0.090	0.316	
		Bonferroni (*Active* vs. Random)	Mean Difference (Group 1 - Group 2)	Std. Error	*p*-value	
		Response	0.056	0.097	0.579	
		No-response	-0.341	0.122	0.024	
	Overlap (T∩E)	RM-ANOVA (Response x Drop type)	F-value	*p*-value	Partial η2	3B.3, bottom right
		Response (within-subjects)	F(1,8) = 20.509	0.002	0.719	
		Drop type (within-subjects)	F(1,8) = 7.443	0.026	0.482	
		Response x Drop type	F(1,8) = 10.263	0.013	0.562	
		Bonferroni (*Active* vs. Random)	Mean Difference (Group 1 - Group 2)	Std. Error	*p*-value	
		Response	-0.182	0.057	0.012	
		No-response	0.036	0.024	0.165	
